# Integrating Network Pharmacology and In Silico Analysis to Explore the Bioactive Compounds Against Gastric Cancer Treatment

**DOI:** 10.7759/cureus.75779

**Published:** 2024-12-16

**Authors:** Smruti P Pradhan, Ayushman Gadnayak, Sukanta Kumar Pradhan, Venkatarao Epari

**Affiliations:** 1 Community Medicine, Siksha 'O' Anusandhan Deemed to be University Institute of Medical Sciences and SUM Hospital, Bhubaneswar, IND; 2 Centre for Biotechnology, Siksha 'O' Anusandhan University, Bhubaneswar, IND; 3 Department of Bioinformatics, Odisha University of Agriculture and Technology, Bhubaneswar, IND

**Keywords:** gastric cancer, gene ontology, in silico approach, molecular docking, network pharmacology

## Abstract

Gastric cancer (GC) has become a major challenge in oncology research, primarily due to its detection at advanced stages. In this study, we identified and validated the pharmacological mechanisms involved in treating gastric cancer using an integrated approach combining network pharmacology, molecular docking, and a dynamic approach. Gastric cancer-related genes were obtained from DisGeNET, Genecard, and Malacard databases, while potential targets of bioactive compounds were predicted using SwissTargetPrediction. Network pharmacology and gene ontology (GO) enrichment analyses were employed to understand the molecular mechanisms of action. This should further be investigated to isolate bioactive compounds that can be used to treat different ailments. Albumin (ALB), B-cell lymphoma 2 (BCL-2), nuclear factor kappa B subunit 1 (NFKB1), hypoxia-inducible factor 1 alpha (HIF1A), and interleukin 6 (IL-6) had a higher expression in gastric cancer than in normal conditions. Top genes were validated by using the GEPIA (Gene Expression Profiling Interactive Analysis) database. Furthermore, the lead compounds dehydroxy-isocalamendiol and spathulenol exhibited the highest binding affinity with NFKB1 and HIF1A (−6.3 and −6 kJ/mol) in the molecular docking study. Enrichment analysis indicated enrichment of these hub targets in the programmed cell death-ligand 1 (PD-L1) checkpoint, phosphatidylinositol 3-kinases/protein kinase B (PI3K-Akt), Ras, and hypoxia-inducible factor-1 (HIF-1) signalling pathways with significant cut-offs of FDR < 0.01 and p < 0.05. Therefore, network pharmacology and molecular docking analyses revealed that dehydroxy-isocalamendiol and spathulenol exert therapeutic efﬁcacy on gastric cancer by multiple targets, NFKB1 and HIF1A, and pathways (MAPK, PD-L1 checkpoint, PI3K-Akt, Ras, and HIF-1 pathways).

## Introduction

In the year 2020, there will be more than one million new cases of gastric cancer (GC) and an estimated 769,000 deaths (equating to one in every 13), placing it fifth in incidence and fourth in mortality globally [1]. The incidence of gastric cancer is ranked fifth, while the fatality rate is ranked third. The five-year survival rate is less than 20% of those diagnosed with gastric cancer will survive the disease [2]. Globally, infection with *Helicobacter pylori*, smoking, and excessive salt consumption are the primary risk factors for GC. Gastrostomy, chemotherapy, and radiation therapy are the most frequently used methods to treat GC [3].

Consequently, novel treatment approaches or effective medications to treat GC are urgently required. Herbal medicine is one of the primary contenders for cancer treatment as it provides a safe, non-toxic, enhanced survival rate and an easily accessible source of therapy. Multiple studies have shown that the risk of GC may decrease using medicinal herbs containing natural components [4]. Phytochemicals in plants are primarily responsible for their medicinal properties [5]. Herbal remedies have several beneficial effects on GC, including reducing cell proliferation and *H. pylori* infection, increasing cell death and autophagy, regulating cell metastases, and demonstrating anti-angiogenic activity, according to prior investigations [6,7].

The present study focused on various critical anti-cancer phytochemicals. Medicinal plants have played an essential role in the treatment of multiple diseases [8]. Many herbs, like *Falcaria vulgaris* Bernh, *Origanum vulgare*, *Cinnamomum zeylanicum*, *Andrographis paniculata*, etc., have been utilised since ancient times due to their health-promoting qualities and a particular position in traditional medicine [9]. Here, we identified the phytochemical components derived from *F. vulgaris* Bernh. (Spathulenol) [10], *Ficus sycomorus* fruits and leaves (Psoralene) [11], *Rubia tinctorum* (dehydroxy-isocalamendiol) [12], *Thymus vulgaris* [13], *O. vulgare* (Thymol) [14], *Khaya senegalensis* (Desr.) A. juss (Linalool) [15], *C. zeylanicum* (2-Propenal, 3-phenyl, 3-phenylpropenal, and cinnamal) [3], *Andrographis paniculate* [16], and *R. tinctorum* (9,12-Octadecadienoic acid (Z, Z) [17]. In addition to treating conditions including gastric cancer, skin disorders, infertility, analgesia, heart disease, and others, the bioactive chemicals found in these plants include antioxidant, antibacterial, anticancer, and antimicrobial characteristics. The apoptotic pathway is a critical mechanism for destroying malignant cancer cells by plant compounds. A defensive mechanism known as apoptosis, or programmed cell death (PCD), is brought on by molecular processes and cell morphological changes, such as chromatin condensation, blabbing, shrinkage, and nuclear fragmentation. The medicinal plant *Acorus calamus* (*A. calamus*) has many health benefits, mainly problems related to the gastrointestinal tract. It has many beneficial properties, like anti-fungal, anti-diabetic, anti-cancer, anti-inflammatory, anti-oxidant, anti-ulcer, pesticidal, cardioprotective, radioprotective, and more [18]. The potential use of plants in the Apiaceae family as prescription drugs, particularly those of the genus *F. vulgaris Bernh.* [10]. These herbs, which have antibacterial properties and are used to treat gastrointestinal disorders, skin conditions, infertility, analgesia, heart disease, and other conditions, are often employed in traditional Iranian medicine [19].

The bioactive compounds spathulenol, psoralene, dehydroxy-isocalamendiol l,2-propenal, 3-phenyl, 3-phenylpropenal cinnamal, 9,12-octadecadienoic acid (Z, Z)-, linalool, and thymol were studied in this study using molecular docking and MD simulation to determine the stable interactions between the treatment targets and the compound itself. The molecular docking analysis yielded important information on the components' orientation and binding affinity inside the active site. MD simulations over a 100-nanosecond trajectory examined its stability and dynamic behaviour. According to the research, certain components may have therapeutic value and potential bioactivity for treating gastric cancer.

## Materials and methods

Data collection and prediction of bioactive components

Bioactive compounds, which have the potential to be anti-cancer treatment targets, were culled from extensively studied scientific articles published throughout the globe. After cross-validation, the SDF structure format was used to save the obtained chemical structures from PubChem [20]. Subsequently, SwissADME was implemented to anticipate potential compounds [21,22]. Briefly, databases were employed to identify targets screened against GC matches. Therapeutic targets linked to GC were collected from the Genecards database [23], the Disgenet database [24], and Malacards [25], with the latter deleting targets with DSI_g less than 0.1 and the former eliminating targets with a relevance score <1 using the keyword ‘gastric cancer’. GC hits were defined as targets that showed up more than twice. The Venn diagram displays the intersection results. Lastly, the intersection targets were visualised using Venny 2.1.0.

Acquiring targets related to compounds

The expected targets of the screened bioactive compounds were determined by inputting the SMILES into the Swiss Target Prediction (STP) databases [26]. The Swiss Target Prediction database's target genes were compared to the three databases' curated common genes to identify them.

Network of compound-target diseases

Cytoscape 3.9.1 tools were used to construct a compound-target disease network to research and display links between disease-associated pathways and plant-based component gene targets. Anti-cancer targets were built after importing the active components and targets using Cytoscape 3.9.1 to display. In this network, nodes represent various elements or goals, while lines show their relationships. A more excellent degree value for a node indicates that it is more important in the network. Essential active ingredients were identified using degree values.

Analysis of protein-protein interaction

To build a protein-protein interaction (PPI) network that includes both direct and indirect protein interactions, the STRING database's enrichment analysis was used to determine the principal target genes formed when two gene datasets interacted [27]. The selection criteria for the STRING database only took Homo sapiens into account, with a confidence value greater than 0.7 serving as the cutoff. Afterwards, the Cytohubba module of Cytoscape was used to display proteins with dense connections - potential anti-cancer treatments centred on these newly identified genes.

Enrichment analysis GO and KEGG

Gene ontology (GO) and the Kyoto Encyclopaedia of Genes and Genomes (KEGG) were used to conduct the functional enrichment analysis of associated therapeutic targets. The biological processes associated with the core therapeutic targets were uncovered by GO enrichment analysis, and the functional pathway annotations of these targets were shown by KEGG enrichment analysis. The DAVID database [28] and SRPlot [29] were used to investigate and visualise three GO keywords: biological function, molecular function, and cellular component. To perform KEGG pathway enrichment analysis, the ShinyGO 0.80 database [30] was employed, and SRPlot was used for visualisation. A significant FDR < 0.01 and p < 0.05 cut-off were utilised to execute the KEGG pathway enrichment analysis. After determining the best route, the KEGG mapper [31] highlighted the specific chemical process along that pathway.

Validation of hub targets

The expression levels of the target genes were evaluated using the GEPIA platform (Gene Expression Profiling Interactive Analysis) [32]. A web-based platform, GEPIA, boasts a vast collection of expression profiles, enabling interactive analysis of gene expression, patient survival data, and gene identification, all of which contribute to disease prognosis and the therapeutic discovery process. Using the STAD (stomach adenocarcinoma) dataset, the mRNA expression levels of the five target genes (ALB, BCL-2, NFKB1, HIF1A, and IL-6) were analysed. The mRNA expression levels were obtained using a log2FC threshold of less than 2, with a p-value of 0.01 as the significance cutoff. A boxplot was used to visualise and compare the expression levels of the target genes between normal and tumour samples. A 95% confidence interval is set for the survival analysis.

Molecular docking

The binding process between target proteins and potential therapeutic ligands is comprehensively comprehended through molecular docking technologies. The UniProt database [33] was first used to validate the hub genes. The crystal structures of the hub target proteins ALB (AF-P02768), BCL2 (AF-P10415), NFKB1 (AF-P19838), HIF1A (AF-Q16665), and IL6 (AF-P05231) were given in PDB format by the RCSB Protein Data Bank [34]. Protein targets were stripped of water, ligands, ions, and heteroatoms. Then, polar hydrogen atoms, gasteiger charges, and Kollman charges were added to create three-dimensional structures. The SDF format ligands, which include 2-Propenal, 3-phenyl, 3-phenylpropenal cinnamal, 9,12-Octadecadienoic acid (Z, Z)-, dehydroxy-isocalamendiol, Linalool, Psoralene, Spathulenol, and Thymol, were retrieved from the PubChem database [20]. SDF files were converted to PDB format using the visualisation tool PyMol. The prankweb web server [35] was used to identify the protein active sites. Bioactive components (ligands) interact with refined protein targets in Autodock Vina. The Pymol visualisation program was employed to convert PDB data for proteins and ligands to PDBQT format. Reproducing visible contacts from the PDB structure and a low root mean square deviation (RMSD) (<3 Å) between the docked ligand and the orientation of the co-crystallised ligand showed that the docking was correct. The BIOVIA Discovery Studio Visualiser application has observed the optimal docking positions.

Molecular dynamic simulation

The Schrödinger suite's Desmond program was used to conduct molecular dynamics simulations (D. E. Shaw Research: Resources) [36]. In this simulation, an orthorhombic box was made using a water-based simple point charge (SPC) solvent model. The model was suitably adjusted, and the salt content stayed constant at 0.15 M. As the constant temperature and pressure (NPT) settings, 310 K and 1.013 bar ambient pressure were used in a 100 ns MD simulation. A semi-isotropic Parrinello-Rahman barostat and a Nose-Hoover thermostat were used, respectively. One thousand frames were obtained by photographing 50 picosecond (ps) intervals throughout the experiment. Following the MD study, the 2000 trajectory snapshots underwent post-MD analysis, which included stability, flexibility, and correlation between molecules.

## Results

Target identification and screening

**Figure 1:**
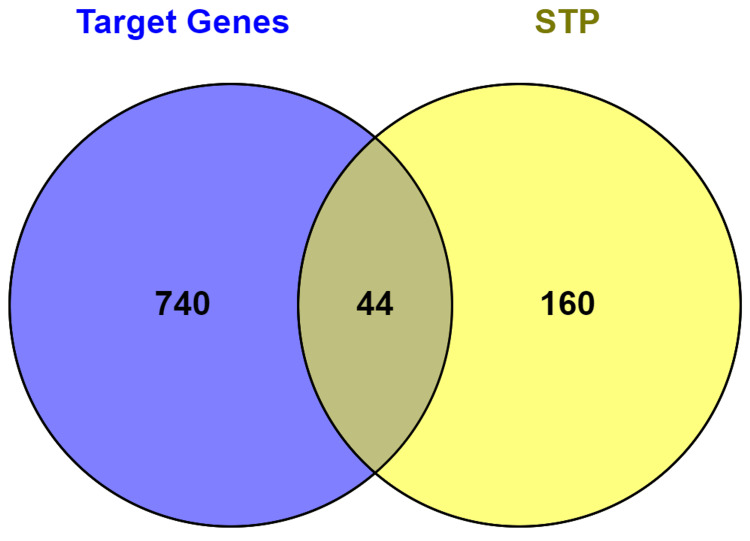
Venn diagram analysis of common target prediction for anti-GC using (GeneCards, Malacards, and DisGeNET) databases and compound-target gene intersection using SwissTargetPrediction (STP).

Gastric cancer-related targets were identified by searching the GeneCards, Malacards, and DisGeNET databases with the terms gastric and stomach cancer. A total of 784 targets associated with GC were identified. In addition, SwissTargetPrediction (STP) was employed to identify 204 targets for anti-cancer therapy. Results showed that 44 genes interacted with bioactive compounds and GC-related targets (Figure 1).

The primary goal of drug-likeness evaluation is to predict possible therapeutic ligands. Based on our findings, bioactive compounds have properties that align with Lipinski's rule of 5. A drug-like molecule must have a polar surface area (PSA) of 140 A2 or less, a molecular weight (MW) of less than 500 g/mol, and an octanol/water partition coefficient (XLogP3) of less than 5. These three factors are essential for drug-ligand interactions to happen. In contrast, there should be no more than ten hydrogen bond acceptors, five hydrogen bond donors, and a rotatable bond of less than 10. The chemical features of the bioactive compounds, as shown in Table 1, are in agreement with Lipinski's rule of 5, indicating that they displayed remarkable drug-like properties.

**Table 1:** This table lists the bioactive constituents identified from anti-cancer based on drug-likeness criteria, including Lipinski's rule of 5, Veber's rule, Egan's rule, and Abbott Bioavailability score. Out of 253 constituents identified, 5 were deemed bioactive as they met the criteria specified.

Sl no.	Compounds	Molecular weight (≤500) (g/likeness mol)	Mlogp (<4.15)	H-bond acceptors (<10)	H-bond donors (<5)	Lipinski	Veber	Egan	Abbott bioavailability score	Drug likeness
1	Spathulenol	220.35	3.67	1	1	Yes	Yes	Yes	0.55	Pass
2	dehydroxy-isocalamendiol	220.35	3.56	1	1	Yes	Yes	Yes	0.55	Pass
3	Psoralene	186.16	1.48	3	0	Yes	Yes	Yes	0.55	Pass
4	Linalool	154.25	2.59	1	1	Yes	Yes	Yes	0.55	Pass
5	Thymol	150.22	2.76	1	1	Yes	Yes	Yes	0.55	Pass

Compound target network analysis

**Figure 2:**
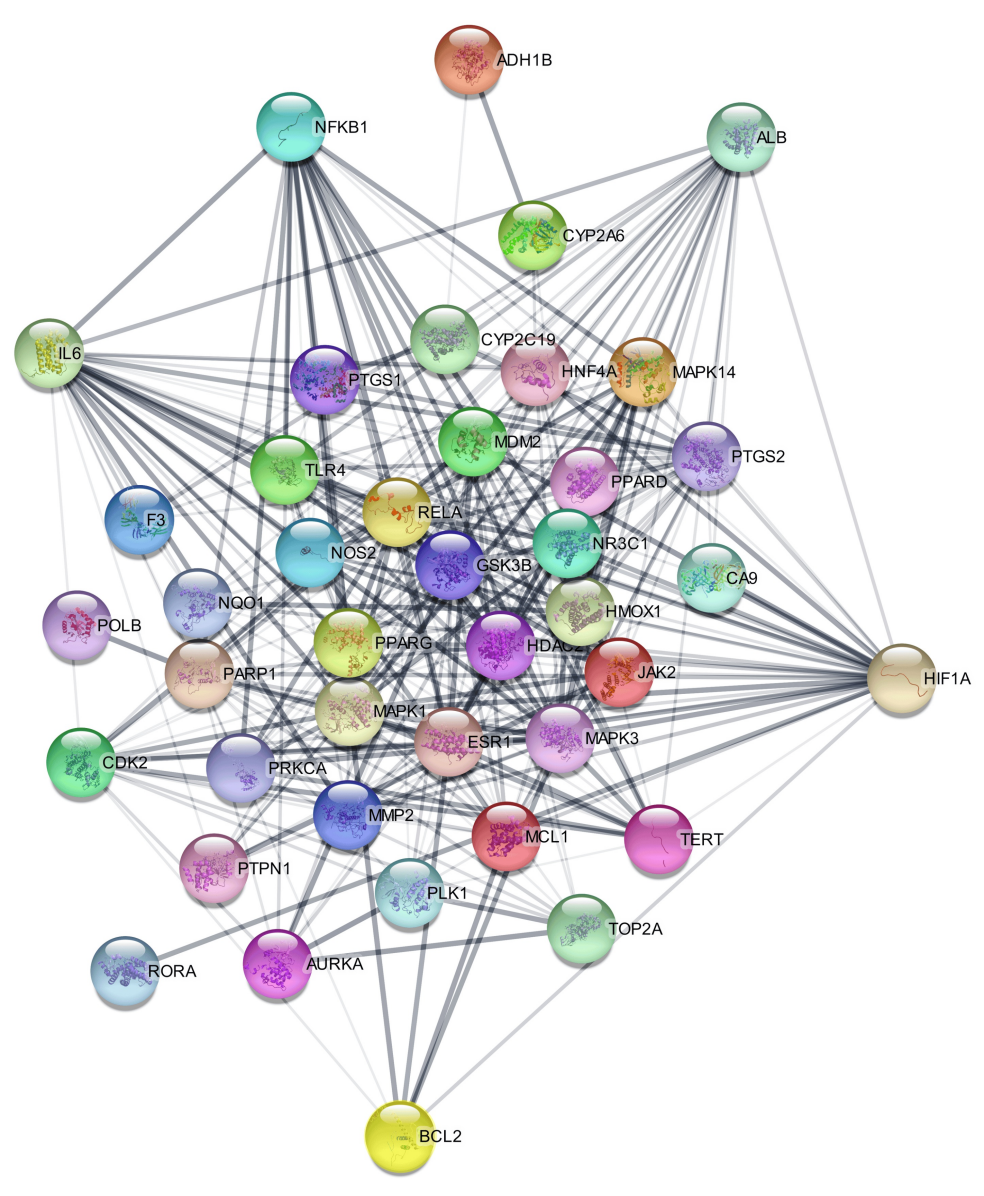
The protein-protein interaction (PPI) network diagram generated from compound targets common intersecting targets. The labeled nodes are known as target proteins for gastric cancer, having 3D structures.

**Figure 3:**
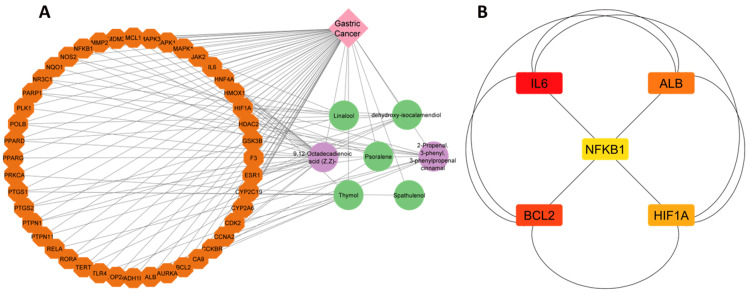
(A) The compound-target disease network illustrates the relationship between compounds and targets that are involved in gastric cancer treatment, illustrating that a single compound (shown in green and purple colour) can target a multitude of genes shown in (orange colour), by using the Cytoscape 3.9.1. (B) Illustration of the top five hub-genes from the protein-protein interaction analysis by using cytoHubba plug-in.

Excluding unrelated nodes, the STRING database was searched for 44 common intersecting targets and filtered to a confidence level of 0.7. Following the selection procedure, the refined targets were imported into the Cytoscape 3.9.1 program, resulting in a PPI network diagram with 44 nodes and 417 edges (Figure 2). As shown in Figure 3A-3B, 35 nodes were identified as interacting through 93 edges within this network. Establishing a pharmacological network helped us understand how one compound may influence several genes. Filtering was implemented according to the ADMET value to identify critical components among the identified elements, i.e., spathulenol, dehydroxy-isocalamendiol, psoralene, linalool, and thymol (Table 2).

**Table 2:** Lists the primary hub compounds discovered in the compound-target disease network designed to treat cancer. Hub chemicals were chosen based on their degree value, which indicated their importance in the network.

Compounds	PubChem ID	Molecular formula	Molecular weight (g/mol)
Spathulenol	92231	C15H24O	220.35
dehydroxy-isocalamendiol	535379	C15H24O	220.35
Psoralene	6199	C11H6O3	186.16
Linalool	6549	C10H18O	154.25
Thymol	6989	C10H14O	150.22
2-Propenal, 3-phenyl, 3-phenylpropenal cinnamal	637511	C9H8O	132.16
9,12-Octadecadienoic acid (Z, Z)-	5280450	C18H32O2	280.4

Protein-protein interaction analysis of intersecting targets

After being chosen, the targets were imported into the Cytoscape 3.9.1 program, which resulted in the construction of a PPI network diagram with 44 nodes and 147 edges (Figure 3A). Subsequently, the Cytohubba plugin was employed to conduct topological screening for protein-protein interaction analysis, focusing on degree centrality. This analysis identified five key proteins: ALB, BCL2, NFKB1, HIF1A, and IL6 (Figure 3B).

GO enrichment and KEGG pathway analysis

**Figure 4:**
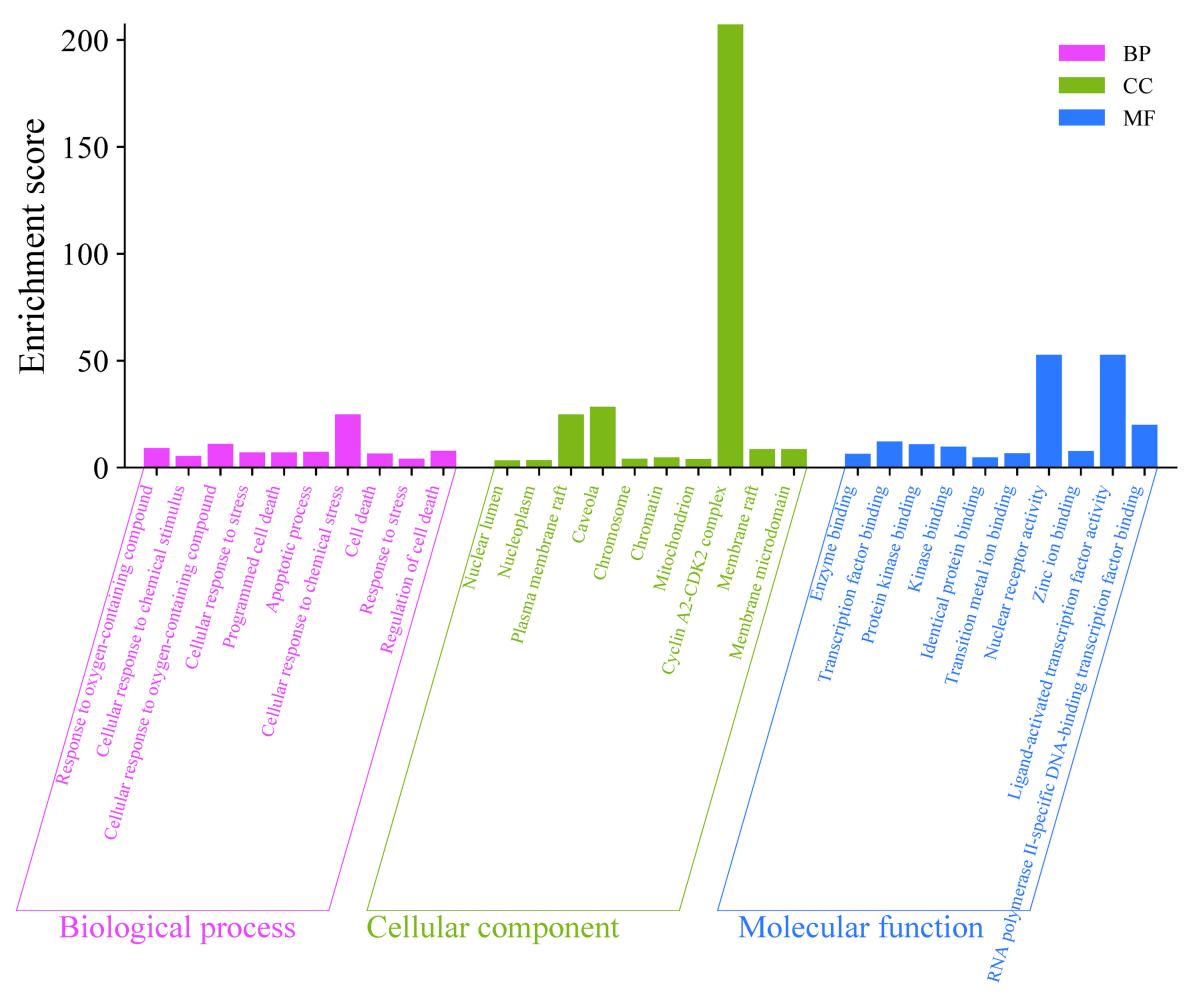
Gene ontology enrichment analysis of 44 bioactive compounds target biological process (BP), cellular component (CC), and molecular function (MF) at p < 0.05 and FDR < 0.01. A bar plot graphic depicts the top 30 functional annotations connected to anti-gastric cancer based on their -logP value.

The primary targets of 44 overlapping genes involved in cellular component (CC), ion (MF), and biological process (BP) were identified as molecular functions by a GO study. Figure 4 shows that the 30 most important words for BP, CC, and MF were found when the thresholds for p < 0.05 and FDR < 0.01, respectively. According to the results, all 44 intersecting targets were associated with biological processes (BP). These processes include, among other things, the response to oxygen-containing compounds, cellular response to stress, programmed cell death, the apoptotic process, cellular response to chemical stress, cell death, response to stress, and regulation of cell death. The core genes enriched with cellular components (CC) were mainly connected to the nuclear lumen, nucleoplasm, plasma membrane raft, caveola, chromosome, chromatin, mitochondrion, cyclin A2-CDK2 complex, membrane raft, and membrane microdomain. In terms of molecular function (MF), the targets were linked to enzyme binding, transcription factor binding, protein kinase binding, kinase binding, identical protein binding, transition metal ion binding, nuclear receptor activity, zinc ion binding, ligand-activated transcription factor activity, and RNA polymerase II-specific DNA-binding transcription factor binding.

**Figure 5:**
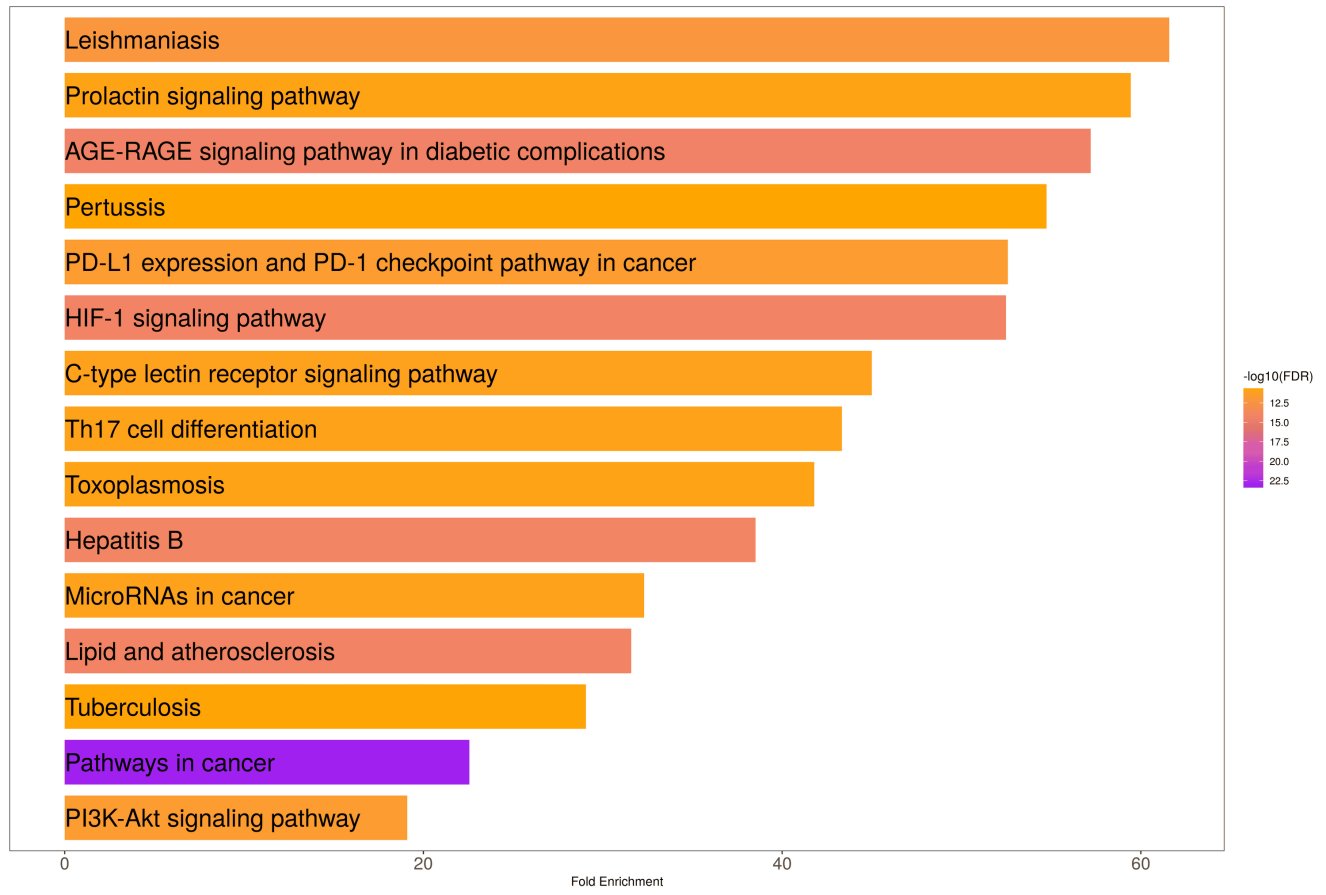
The pathway analysis focuses on important pathways related to gastric cancer discovered in KEGG databases at p < 0.05 and FDR < 0.01. A bar plot graphic depicts the most significant pathways emphasizing their involvement in promoting targets for anti-gastric cancer compounds.

**Figure 6:**
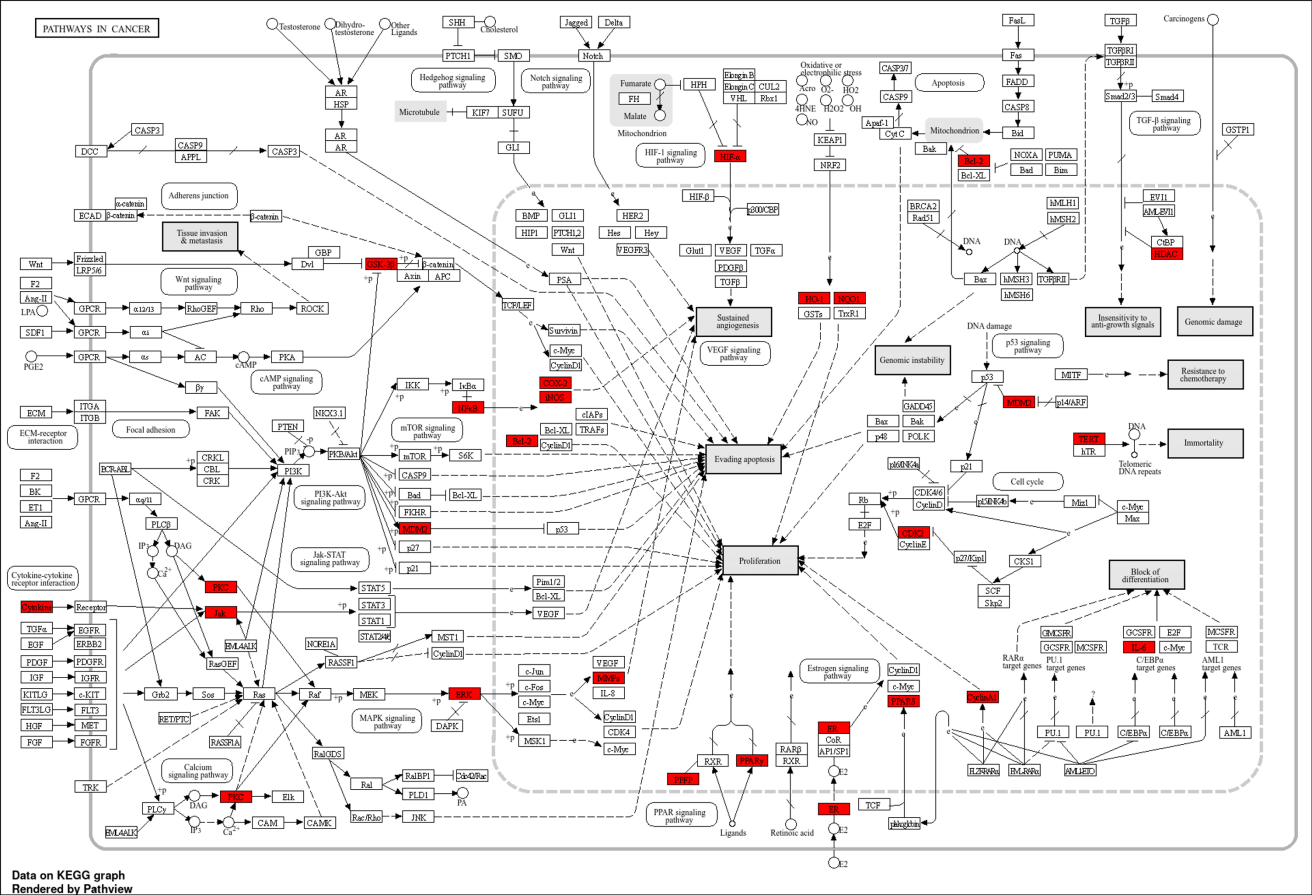
The pathway analysis focuses on important target genes related to cancer discovered in KEGG databases, numerous components, and key actors engaged in these pathways, emphasizing their involvement in promoting proinflammatory responses and prospective targets.

Figure 5 shows that 15 signalling pathways were found by KEGG enrichment analysis with a p < 0.05 and FDR < 0.01 criterion. A total of 3131 significant genes were associated with these top 15 signalling pathways detailed in supplementary Table 12. Among all the pathways, leishmaniasis is the most significant pathway. After that, the prolactin signalling pathway and AGE-RAGE signalling pathways in diabetic complication pathways are highly significant (Figure 5). The attention next shifted to the pathways in cancer; the CDK2, NQO1, ESR1, GSK3B, HDAC2, HIF1A, HMOX1, IL6, JAK2, MDM2, MMP2, NFKB1, NOS2, PPARD, PPARG, PRKCA, MAPK1, MAPK3, PTGS2, BCL2, RELA, TERT, and CCNA2 genes are highly associated with this pathway (Figure 6). The investigation demonstrated the involvement of various components, evading apoptosis, proliferation, sustained angiogenesis, immortality, and genomic instability. In the HIF-1 signalling pathway, the NF-kB signalling pathway, HIF-α, COX2, and iNOS are associated. BCL2 and CDK2 are involved in proliferation.

Validation of hub targets

**Figure 7:**
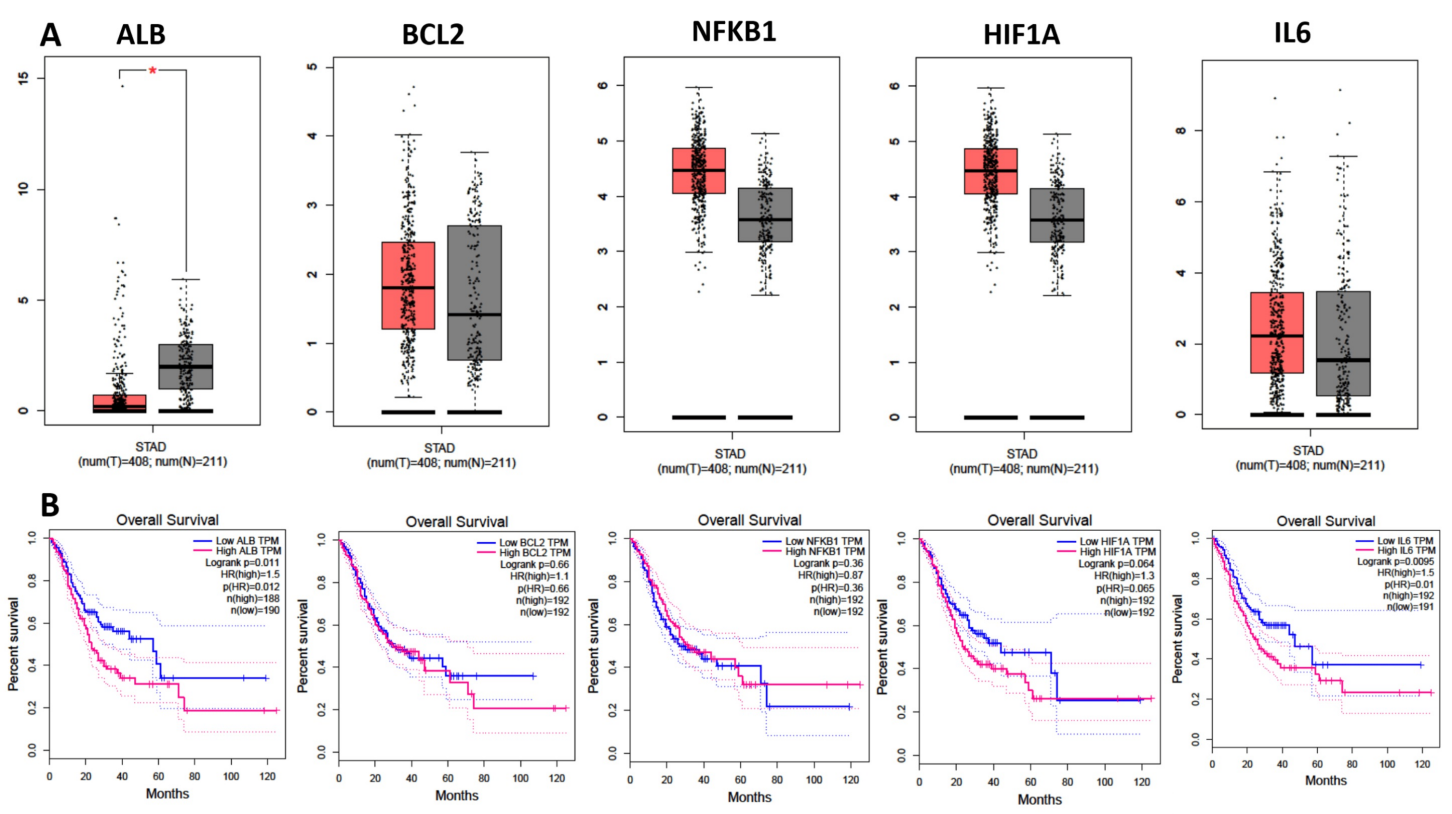
mRNA expression level, pathological stage, and OS of hub targets. (A) Box plots showing the mRNA expression levels of (a) ALB, (b) BCL-2, (c) NFKB1, (d) HIF1A, and (e) IL-6. Red represents a tumour, and grey represents normal. (B) The line charts show the OS of hub targets. The survival curve comparing the patients with high (red) and low (blue) expression in GC.

The mRNA expression levels of the identified targets were examined using the GEPIA database based on the findings from the system pharmacology analysis. The study analysed the mRNA expression levels of ALB, BCL-2, NFKB1, HIF1A, and IL-6 in 408 stomach adenocarcinoma samples alongside non-tumour gastric tissues. The findings revealed that the expression of NFKB1 and HIF1A genes was significantly high, and IL-6 expressed the same between the stomach adenocarcinoma tissues compared to normal tissues (Figure 7A). The Kaplan-Meier survival analysis demonstrated that all these hub genes were significantly high in prognostic value (P < 0.05), as shown in Figure 7B.

Molecular docking and molecular dynamics simulation

**Figure 8:**
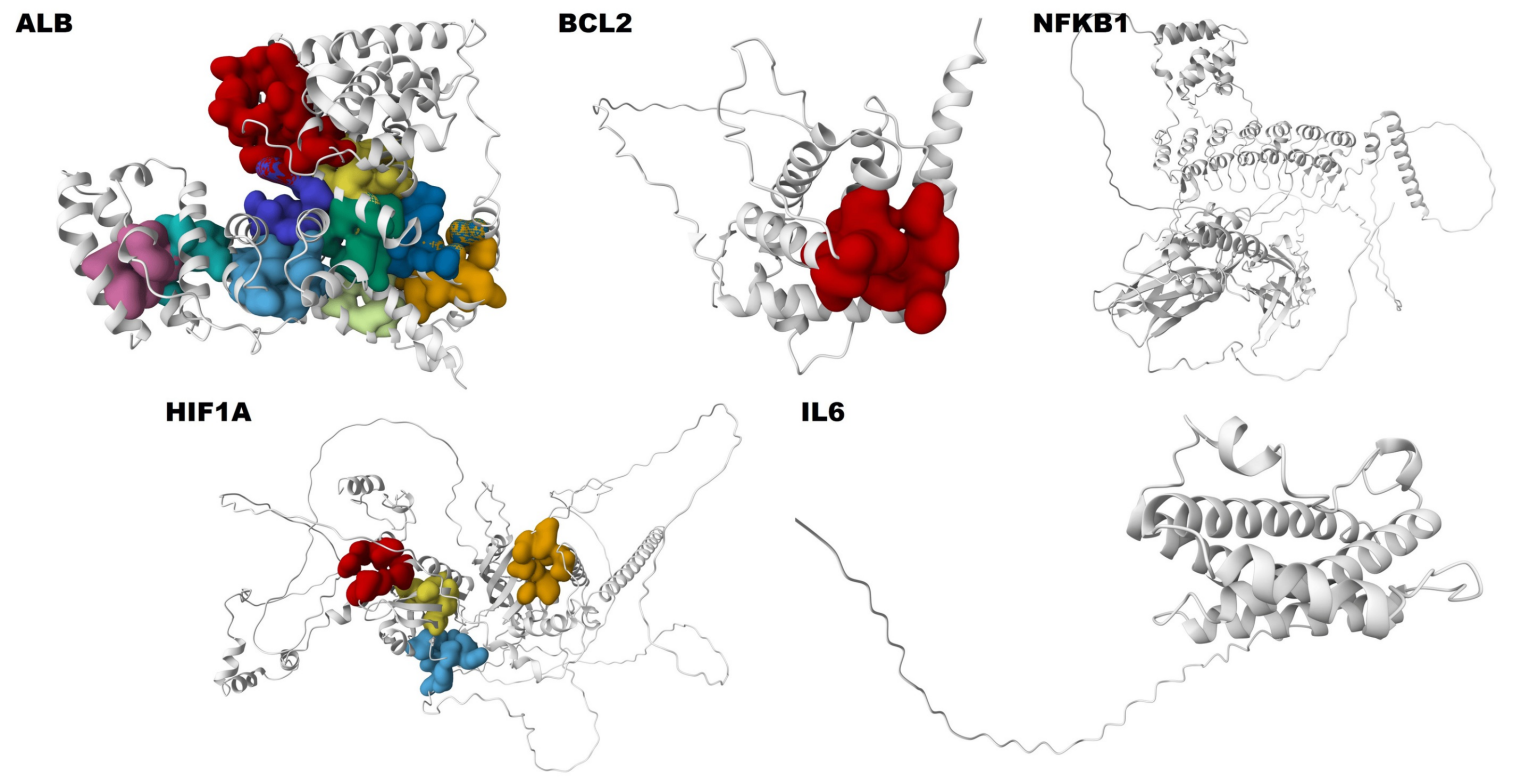
The binding of ligands to the active sites of target proteins, providing valuable insights into the interaction between the ligands and the protein receptors spathulenol-ALB, spathulenol-BCL2, dehydroxy-isocalamendiol-NFKB1, spathulenol-HIF1A, and dehydroxy-isocalamendiol-IL6.

Using molecular docking, the binding affinities of bioactive components to hub target genes were established. Spathulenol, dehydroxy-isocalamendiol, psoralene, linalool, and thymol were chosen as active ingredients, along with five target proteins: ALB, BCL2, NFKB1, HIF1A, and IL6, based on their high degree scores from network screening. Utilising AutoDock Vina software, the molecular docking technique was carried out; a greater negative docking score indicates a stronger binding affinity between the ligand and protein, explained in detail in Table 3. Figure 8 shows the target proteins' active sites of the active ingredients and co-crystallized ligands/inhibitors.

**Table 3:** Docking binding affinities of active constituents with the active sites of target proteins ALB, BCL2, NFKB1, HIF1A, and IL6. The free binding energy (in kcal/mol) of each constituent with its respective target protein is provided, along with a description of the residues involved in the binding interface.

Proteins with pdb id	Compounds	Binding affinity (kcal/mol)
ALB	Spathulenol	−7.3
	Psoralene	−7.3
	Dehydroxy-isocalamendiol	−7.1
	Thymol	−6.3
	Linalool	−5
BCL2	Spathulenol	−6.5
	Psoralene	−6.3
	Dehydroxy-isocalamendiol	−6.2
	Linalool	−5.4
	Thymol	−5.1
NFKB1	Dehydroxy-isocalamendiol	−6.3
HIF1A	Spathulenol	−6
	Psoralene	−5.9
	Dehydroxy-isocalamendiol	−5.7
	Thymol	−5.2
	Linalool	−4.9
IL6	Dehydroxy-isocalamendiol	−5.6

**Figure 9:**
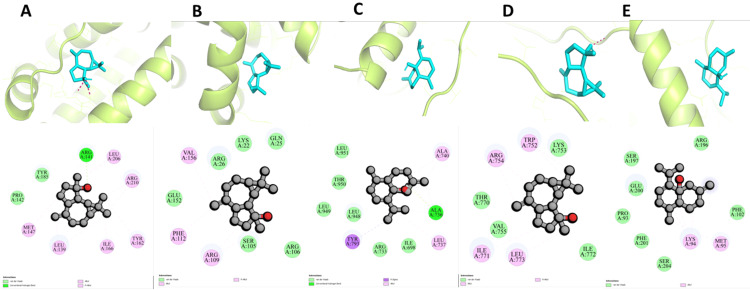
The molecular docking results of 2D and 3D images of active constituents with target protein (A) spathulenol-ALB, (B) spathulenol-BCL2, (C) dehydroxy-isocalamendiol-NFKB1, (D) spathulenol-HIF1A, and (E) dehydroxy-isocalamendiol-IL6. Each subfigure illustrates the interaction between an active constituent and its respective target proteins. The interactions are depicted to demonstrate the binding affinity, with details of the residues involved in the binding interface. The hydrogen bonds are shown as the red dots.

Spathulenol exhibited the highest binding affinity with ALB, showing a free binding energy of −7.3 kcal/mol. This interaction primarily involved conventional hydrogen bonds with ARG141, van der Waals forces with TYR185 and ALA272, as well as alkyl and pi-alkyl interactions with LEU206, ARG210, TYR162, ILE166, LEU139, and MET147 residues of ALB (Figure 9A). Spathulenol demonstrated the second-highest good binding affinity with BCL2, with a free binding energy of −6.5 kcal/mol. This interaction involved van der Waals interactions with GLU152, ARG26, LYS22, GLN25, SER105, and ARG106, as well as alkyl and pi-alkyl interactions with VAL156, PHE112, and ARG109 residues of BCL2 (Figure 9B). The binding affinity of dehydroxy-isocalamendiol with NFKB1 has a free binding energy of -6.3 kcal/mol. This interaction involved a conventional hydrogen bond with ALA736, van der Waals interactions with LEU951, THR950, LEU949, LEU948, ARG733, and ILE698, as well as pi-sigma and alkyl interactions with TYR793 and LEU737 and ALA740 residues of NFKB1 (Figure 9C). Similarly, spathulenol has a binding affinity with HIF1A, with a free binding energy of −6 kcal/mol. This interaction involved van der Waals interactions with THR770, VAL755, LYS753, and ILE772, as well as alkyl and pi-alkyl interactions with ARG754, TRP752, ILE771, and LEU773 residues of HIF1A (Figure 9D). Furthermore, dehydroxy-isocalamendiol exhibited the highest binding affinity with IL-6, with a free binding energy of −5.6 kcal/mol. This interaction involved van der Waals interactions with ARG196, SER197, GLU200, PRO93, PHE201, SER204, ARG196, and PHE102 alkyl LYS94 and MET95 residues of IL6 (Figure 9E).

**Figure 10:**
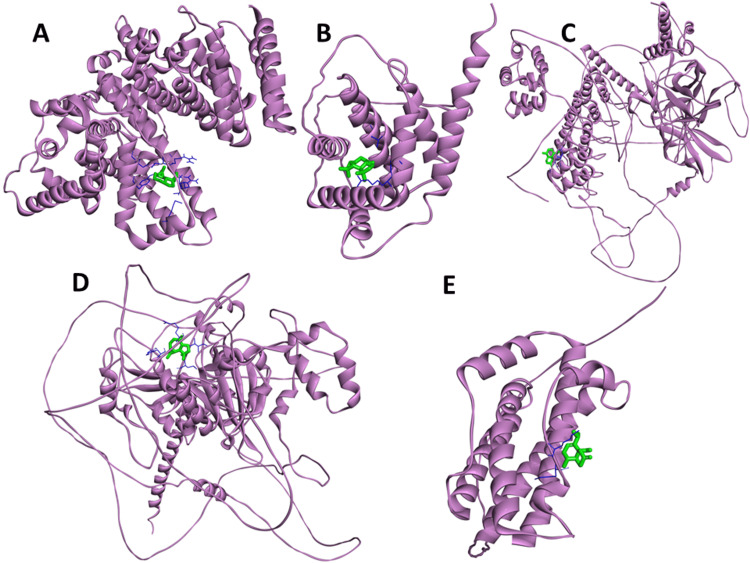
The orientation of proteins within the ligand-binding pockets of (A) spathulenol-ALB, (B) spathulenol-BCL2, (C) dehydroxy-isocalamendiol-NFKB1, (D) spathulenol-HIF1A, and (E) dehydroxy-isocalamendiol-IL6 are depicted in the structural superimposed view of magenta (protein) and lime green (ligand) snapshots.

Although molecular docking is a fast and accurate method for determining ligand-protein binding at the active site of a protein (Figure 8), it does not consider that the protein and ligand undergo conformational stability changes throughout the interaction. To evade this problem, we used molecular dynamics (MD) simulations of the docked complex to examine its stability, conformational changes, and compatibility with the ligand in greater detail. Figure 10 displays the top-ranked cluster (MD trajectories) and docked conformation structures, which both reveal that the ligand prefers to remain in the same binding pocket with small orientation changes. This confirms the accuracy of the docking approach used in this study.

**Figure 11:**
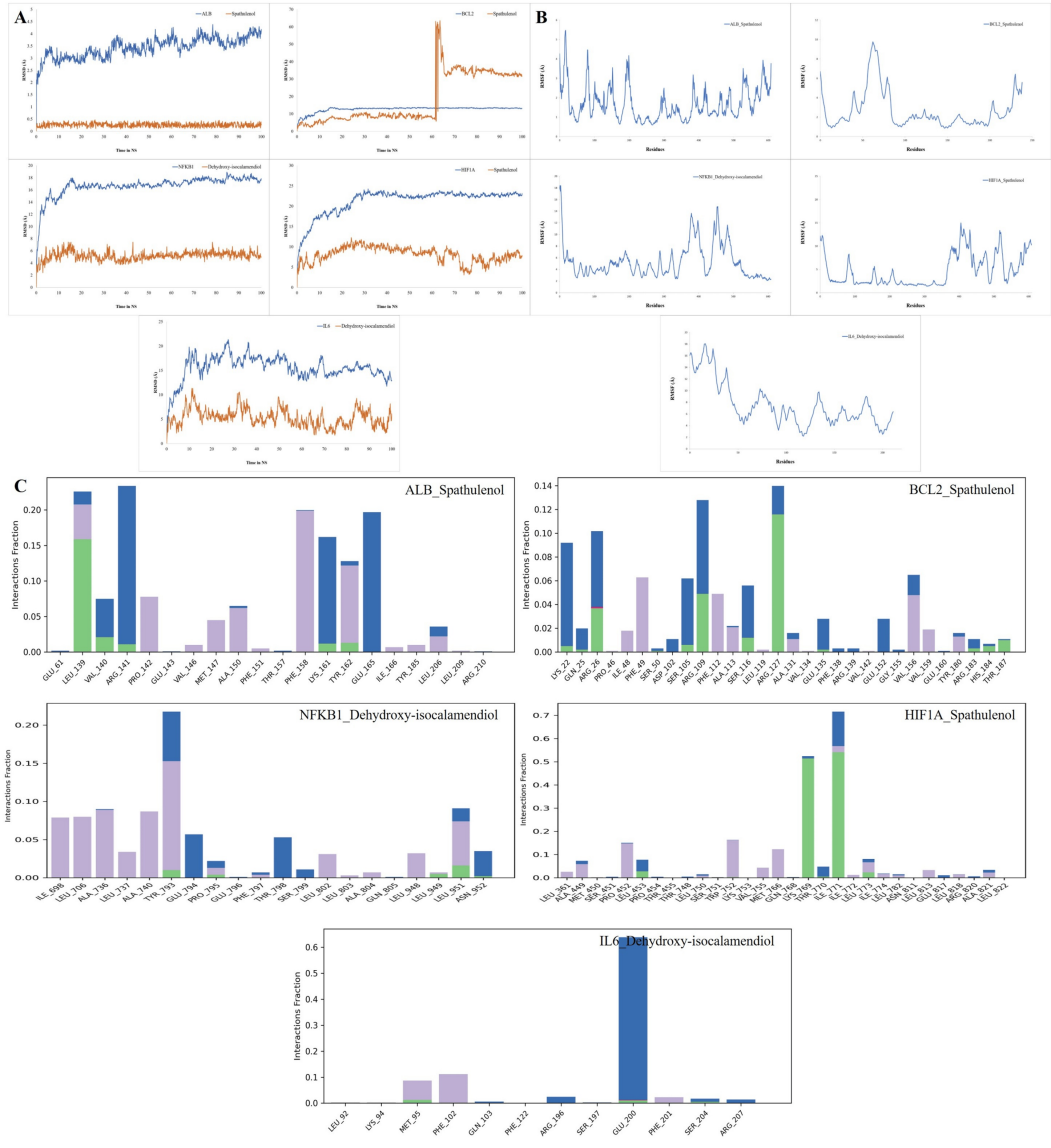
Molecular dynamics (MD) simulations were used to determine conformational changes, stability, and compatibility of protein-ligand complexes. (A) RMSD plots for the spathulenol-ALB, spathulenol-BCL2, dehydroxy-isocalamendiol-NFKB1, spathulenol-HIF1A, and dehydroxy-isocalamendiol-IL6 complex show changes over time, stabilising around 4 Å. (B) RMSF analysis shows low variability of active site residues (~1.5 Å) compound target complexes. (C) The analysis includes hydrogen bonds, hydrophobic contacts, water bridges, and ionic interactions, and consistent bonding is observed throughout the simulations.

However, it does not consider that the protein and the ligand both undergo changes in conformational stability during the interaction. By conducting 100 ns MD simulations on the top-ranked docked complex against the apoprotein, we examined backbone RMSD, root mean square fluctuation (RMSF), and protein-ligand interactions. Based on Figure 11A, the protein-ligand RMSD plots for the ALB (AF-P02768-F1)-Spathulenol complex showed that both protein RMSD exceeded 2 Å, increased to 3.5 Å, and then stabilised around 3 Å up to 35 ns, and the ligand stabilised at 0.3 Å. Little fluctuations were demonstrated at five ns, 24 ns, 35 ns, 52 ns, 60 ns, 72 ns, and 78 ns. After 85 ns, the protein was stable. In the BCL2 (AF-P10415)-Spathulenol complex, the protein RMSD stabilised up to 60 ns around 10 Å, but a major fluctuation showed at 63 ns and then stabilised around 35 Å. The ligand RMSD stabilised at 10 Å. The protein-ligand RMSD plots for the NFKB1 (AF-P19838)-dehydroxy-isocalamendiol complex showed that both protein and ligand are stable at 100 ns around 18 Å and 6 Å. The highest variation of around 18 Å was seen in the root-mean-square fluctuation (RMSF) analysis, which is attributed to the flexibility of the N- and C-terminal loop residues. The protein-ligand RMSD plots for the (HIF1A) AF-Q16665-spathulenol complex showed that protein and ligand are stable at 100 ns around 23 Å and 9 Å. The highest variation of around 16 Å was seen in the RMSF analysis. The protein-ligand RMSD plots for the IL6 (AF-P05231)-dehydroxy-isocalamendiol complex showed that protein RMSD had major fluctuations starting at ten ns, 25 ns, 35 ns, 40 ns, 50 ns, 68 ns, 80 ns, 85 ns, 90 ns, and 95 ns, and ligand RMSD fluctuations starting at ten ns, 30 ns, 35 ns, 42 ns, 45 ns, 50 ns, 65 ns, 70 ns, 78 ns, 85 ns, and 90 ns, and are stable at 100 ns around 15 Å and 10 Å.

In Figure 11B, the residues of ALB (AF-P02768-F1)-spathulenol showed many RMSF fluctuations at 6 Å throughout the simulation. The residues of BCL2 (AF-P10415)-spathulenol showed high fluctuation at the initial and end points. The residues of NFKB1 (AF-P19838)-dehydroxy-isocalamendiol fluctuated at 18 Å, 14 Å, and 16 Å but were stable at the end around 4 Å. The residues of (HIF1A) AF-Q16665-Spathulenol were initially stable at around 5 Å and fluctuated at 15 Å at the end. The residues of IL6 (AF-P05231)-dehydroxy-isocalamendiol showed many fluctuations throughout the simulation.

In Figure 11C, the X-axis shows residues that aid interaction, while the Y-axis shows interaction percentages, including hydrogen bonds, hydrophobic interactions, and water bridges. Through protein-ligand contact analysis, it was found that residues like LEU139, VAL140, ARG141, LYS161, and TYR162 were responsible for the hydrogen bond interaction at 0.01 ns to 0.15 ns, the hydrophobic bond LEU139, PRO142, VAL146, MET47, ALA150, PHE151, PHE158, TYR162, ILE166, TYR185, and LEU206, and the greatest water bridging was LEU139, VAL140, ARG141, LYS161, TYR162, GLU165, and LEU206 were responsible for the interaction of the ALB (AF-P02768-F1)-spathulenol. In the BCL2 (AF-P10415)-spathulenol, it was found that residues like LYS22, GLN25, ARG26, SER50, SER105, ARG109, SER116, ARG127, GLU135, ARG183, HIS184, and THR187 were responsible for the hydrogen bond interaction at 0.01 ns to 0.11 ns, the hydrophobic bond PRO46, ILE48, PHE49, PHE112, ALA113, LEU119, ALA131, VAL134, VAL142, 156, 159, and TYR 180, and the ionic bond at ARG26. The greatest water bridging was LYS22, GLN25, ARG26, SER50, ASP102, SER105, ARG109, ALA113, SER16, ARG127, ALA131, GLU135, PHE138, ARG139, GLU152, GLY155, VAL156, GLU160, TYR180, ARG83, HIS184, and THR187. Hydrogen bonds, hydrophobic contacts, and water bridges were analysed in the NFKB1-dehydroxy-isocalamendiol interaction, showing minor fluctuation during simulation. Through protein-ligand contact analysis, it was found that residues like TYR793, GLU794, PRO795, PHE797, THR798, SER799, LEU951, and ASN952 were responsible for the water bridging at ns, and the residues like ILE698, LEU706, ALA736, LEU737, ALA740, TYR793, PRO795, LEU802, LEU803, ALA804, LEU948, and LEU951 were responsible for hydrophobic contacts with dehydroxy-isocalamendiol, among which TYR793, PRO795, LEU949, ASN952, and LEU951 were the hydrogen bond ones at 0.01 ns to 0.03 ns fractions of the interaction time, respectively. In HIF1A-Spathulenol, it was found that residues like LEU453, LYS769, THR770, ILE771, and LEU773 were responsible for the hydrogen bond interaction at 0.01 ns to 0.6 ns, the hydrophobic bond LEU361, ALA449, PRO452, LEU750, TRP752, VAL755, MET766, ILE771, ILE772, LEU773, ILE774, LEU782, LEU813, LEU818, and ALA821, and the greatest water bridging was ALA449, SER451, PRO452, LEU453, PRO454, THR748, LEU750, LYS769, THR770, ILE771, LEU773, ILE774, LEU782, GLU817, ARG820, and ALA821 were responsible. In IL6 (AF-P05231)-dehydroxy-isocalamendiol, it was found that residues like MET95, GLU200, and SER204 were responsible for the hydrogen bond interaction, the hydrophobic bond LEU92, MET95, PHE102, PHE201, ionic bond at GLU200, and water bridging were LYS94, GLU103, ARG196, SER197, GLU200, SER204, and ARG207.

## Discussion

Various plants include therapeutic chemicals that have anti-cancer properties, according to several research studies [9,37]. The essential oil of *A. calamus* has therapeutic effects due to the harmonious interaction of its different constituents. Haghighi et al. reveal that the methanolic and ethanolic extracts and essential oil of *A. calamus* significantly inhibited human gastric cancer cell growth, and the effects of these extracts varied with dosage and time. It possesses anti-cancer properties for anti-cancer drugs to inhibit the growth of tumours [12]. The considerable antioxidant effect of *F. vulgaris*, attributed to compounds such as alkaloids, anthraquinones, flavonoids, phenols, saponins, steroids, and tannins, has already been examined [38]. Spathulenol is the most volatile bioactive compound of *F. vulgaris*. Spathulenol has been proposed as a promising option for combating drug resistance in cancer therapy [39]. The anti-cancer nature of bioactive compounds was evaluated utilising the Lipinski rule of 5 [40], Veber [41], and Egan [42] calculations, identifying ten compounds with potential pharmaceutical importance. The use of public datasets for subsequent target predictions has uncovered many targets linked to different biological processes. Notably, the research on similarities identified 44 shared targets associated with anti-cancer treatments. When comparing these data with earlier studies, many articles point out the pharmacological effects of certain compounds discovered in gastric cancer (exiting). In this context, the anti-cancer effects of spathulenol [10], psoralene [11], dehydroxy-isocalamendiol [12], thymol [13,14], and linalool [15] have been the subject of much research.

Our research on GO enrichment and network pharmacology elucidates critical molecular pathways involved in gastric cancer. PPI analysis revealed five important proteins, such as ALB, BCL-2, NF-κB, HIF1A, and IL6, from 44 target proteins of gastric cancer according to statistical significance. According to the highest significant level, IL6 comes first in number, then BCL-2, ALB, HIF1A, and NF-κB. These findings were consistent with earlier studies on gastric cancer-related pathways. An independent predictor of malignant tumours was serum albumin. The five-year survival rate of GC patients positively correlated with serum albumin. According to relevant studies, low albumin levels are associated with a poor outcome in GC. The prognosis of GC patients was worse for those with low albumin levels than those with high levels, while albumin level was not a standalone predictor of prognosis [43-46].

Despite cancer, several diseases like rheumatoid arthritis, haemorrhage, and ischaemia use albumin as a biomarker. It plays a crucial role in tumour development, driven by the intense nutrient demand of cancer cells. This role presents a promising approach that could enhance the effectiveness of cancer therapy. Overexpression has been shown to drive cell proliferation and stimulate tumour growth [47]. Over the last decade, extensive studies have been conducted on the function of proteins belonging to the BCL-2 family in controlling cell death, tumour development, and cellular reactions to anticancer treatment. These members display actions that promote or inhibit cell death [48]. As such, a new therapy regimen based on tumour aetiology involves inhibiting the BCL-2 anti-apoptotic protein from overcoming the resistance of tumour cells to apoptosis [49]. BCL-2 family proteins have emerged as potential targets for anticancer medications due to their diverse roles in cancer [50]. Overexpression of BCL-2 promotes tumour growth and chemoresistance [51].

The ubiquitous NF-B transcription factor family controls a wide range of biological processes, including cell differentiation, proliferation, survival, and, most importantly, inflammation and immune responses [52]. One fundamental reason for the development of GC is the dysregulation of NF-κB activation. Prior studies have shown that gastric carcinogenesis is affected by the activation of NF-κB. The stimulation increases the activation of genes associated with cell proliferation, genetic instability, treatment resistance, metastasis, and suppression of apoptosis [53,54]. In gastric cancer, hypoxia and HIF-1α play significant roles in tumour growth and chemoresistance. HIF-1α overexpression was more prevalent in diffuse-type gastric cancers than intestinal-type gastric tumours [55]. HIF-1α expression is strongly linked to aggressive tumour phenotype and poor prognosis in GC. A meta-analysis of nine studies (1103 subjects) found that half of GC patients have HIF-1α-expressing tumours associated with lower five-year survival, deeper invasion, higher lymphatic/vascular invasion risk, and advanced TNM stage [56].

Interleukin-6 is a secretory protein that is particular to cancer-associated fibroblasts (CAFs) and plays a role in the dynamic interaction between tumour cells and the microenvironment that is necessary for tumour development, invasion, and metastasis [57]. IL-6 is highly expressed in GC and plays a significant role in promoting several tumour hallmarks. Research indicates that IL-6 boosts the proliferation and invasiveness of stomach cancer cell lines, while its overexpression in mice triggers the development of multiple carcinomas [58].

In KEGG pathway analysis, programmed death-ligand 1 (PD-L1) expression and PD-1 checkpoint, hypoxia-inducible factor-1 (HIF-1), pathways in cancer, and PI3K/Akt signalling pathways are very crucial in gastric cancer [59-61]. Activation of the Ras/MAPK and PI3K/Akt/mTOR pathways by growth factor receptors can increase HIF-1α expression [62]. In gastric cancer, targeting the PI3K/AKT pathway may be useful to suppress HIF-1α or improve the effectiveness of HIF-1α inhibitors. Since the PI3K/AKT/mTOR/HIF-1α axis drives tumour-promoting inflammation in H. pylori- and EBV-associated gastric cancer, agents that target this pathway may also inhibit the development of premalignant gastric lesions from completing malignancy [56]. MicroRNAs in cancer are associated with mechanisms in gastric cancer development [63].

Strong relationships between the targets and many biological processes, molecular activities, and cellular components linked with cancer were discovered by the GO enrichment analysis. Maintaining the correct structure and operation of the digestive system in the body depends critically on PCD. According to recent studies, H. pylori infection causes aberrant activation of many PCD signalling pathways. Therapy-related malfunction of PCD is believed to have a role in the development of gastric cancer and to impede its therapy [64].

The molecular docking and molecular dynamics simulation results reveal the interactions between bioactive components and key target proteins linked to gastric cancer. Our research provided insight into the potential processes behind anti-cancer benefits by assessing its binding affinity and stability. Active components like spathulenol and dihydroxy-isocalamendiol show strong binding affinities with target proteins like NFKB1 and HIF1A. The study found that bioactive compounds may possess anti-cancer benefits through hydrogen bonding, van der Waals interactions, and hydrophobic contacts, aligning with previous research on natural substances' anti-inflammatory properties. Recent research shows that NFKB1 and HIF1A have been used as versatile drug targets for malignant cells. In GC, NFKB1 and HIF1A are dysregulated and often involved in tumour growth, proliferation, tumour-promoting inflammation, chemoresistance, and cell differentiation.

HIF-1α is crucial in all cancer hallmarks, particularly in hypoxia-induced angiogenesis and resistance to apoptosis in gastric cancer. HIF-1α may contribute to the poor response to immunotherapies or angiogenesis inhibitors [56,65]. Clinical and experimental studies have demonstrated that certain anti-cancer therapies can be enhanced by pharmacologically inhibiting NF-κB, leading to increased cell death. Numerous biological functions, including angiogenesis, evasion of apoptosis, and proliferation, are regulated by NF-κB. In GC patients, NFKB1 is a promising biomarker for diagnosis and therapeutic approach [66].

This research used an in silico analysis to study anti-gastric cancer treatment, which may not fully represent the complex responses. Further in vivo and in vitro studies are needed to validate the findings and assess the in vivo efficacy of anti-gastric cancer. Additionally, research is required to understand the detailed mechanisms underlying its effects, including the role of other signalling pathways and their interactions. And develop new treatment strategies.

## Conclusions

The study offers insights into the anti-cancer properties and molecular mechanisms of bioactive compounds. Chemical profiling, bioactivity screening, and target prediction analyses to identify key bioactive compounds in anti-cancer and their potential pharmacological relevance. The analysis of network pharmacology, KEGG pathway, and GO enrichment analysis revealed the involvement of crucial inflammatory mediators and pathways, aligning with previous research. The study uses molecular docking and molecular dynamics simulation to identify the primary active ingredients in bioactive constituents spathulenol and dehydroxy-isocalamendiol, which may be responsible for their anti-cancer effects. The strong binding affinities between active constituents and target proteins indicate the potential anti-cancer agent. The study suggests that bioactive constituents could potentially be a viable anti-cancer treatment for gastric cancer.

## Appendices

**Table 4:** Summarize important genes related to gastric cancer pathways discovered in KEGG databases.

Enrichment FDR	nGenes	Pathway Genes	Fold Enrichment	Pathway	Genes
6.19E-09	9	76	22.01017	Leishmaniasis	MAPK14 JAK2 NFKB1 NOS2 MAPK1 MAPK3 PTGS2 RELA TLR4
7.09E-08	8	70	21.24156	Prolactin signaling pathway	MAPK14 ESR1 GSK3B JAK2 NFKB1 MAPK1 MAPK3 RELA
3.06E-10	11	100	20.445	AGE-RAGE signaling pathway in diabetic complications	MAPK14 F3 IL6 JAK2 MMP2 NFKB1 PRKCA MAPK1 MAPK3 BCL2 RELA
1.01E-07	8	76	19.56459	Pertussis	MAPK14 IL6 NFKB1 NOS2 MAPK1 MAPK3 RELA TLR4
2.25E-08	9	89	18.7952	PD-L1 expression and PD-1 checkpoint pathway in cancer	MAPK14 HIF1A JAK2 NFKB1 MAPK1 MAPK3 PTPN11 RELA TLR4
5.35E-10	11	109	18.75688	HIF-1 signaling pathway	HIF1A HMOX1 IL6 NFKB1 NOS2 PRKCA MAPK1 MAPK3 BCL2 RELA TLR4
7.14E-08	9	104	16.08435	C-type lectin receptor signaling pathway	MAPK14 IL6 MDM2 NFKB1 MAPK1 MAPK3 PTGS2 PTPN11 RELA
9.01E-08	9	108	15.48864	Th17 cell differentiation	MAPK14 HIF1A IL6 JAK2 NFKB1 MAPK1 MAPK3 RELA RORA
1.04E-07	9	112	14.93547	Toxoplasmosis	MAPK14 JAK2 NFKB1 NOS2 MAPK1 MAPK3 BCL2 RELA TLR4
1.58E-09	12	162	13.76768	Hepatitis B	CDK2 MAPK14 IL6 JAK2 NFKB1 PRKCA MAPK1 MAPK3 BCL2 RELA TLR4 CCNA2
1.52E-07	10	161	11.54433	MicroRNAs in cancer	HDAC2 HMOX1 MCL1 MDM2 NFKB1 PRKCA MAPK1 MAPK3 PTGS2 BCL2
2.04E-09	13	214	11.29078	Lipid and atherosclerosis	MAPK14 CYP2A6 GSK3B IL6 JAK2 NFKB1 PPARG PRKCA MAPK1 MAPK3 BCL2 RELA TLR4
3.49E-07	10	179	10.38344	Tuberculosis	MAPK14 IL6 JAK2 NFKB1 NOS2 MAPK1 MAPK3 BCL2 RELA TLR4
3.50E-14	23	530	8.06578	Pathways in cancer	CDK2 NQO1 ESR1 GSK3B HDAC2 HIF1A HMOX1 IL6 JAK2 MDM2 MMP2 NFKB1 NOS2 PPARD PPARG PRKCA MAPK1 MAPK3 PTGS2 BCL2 RELA TERT CCNA2
3.49E-07	13	354	6.825501	PI3K-Akt signaling pathway	CDK2 GSK3B IL6 JAK2 MCL1 MDM2 NFKB1 PRKCA MAPK1 MAPK3 BCL2 RELA TLR4
